# Supplementary material for: Attractiveness and distinctiveness between speakers' voices in naturalistic speech and their faces are uncorrelated
Source: R Soc Open Sci. 2020 Dec 9;7(12):201244. doi: 10.1098/rsos.201244 (PMC7813223; doi:10.1098/rsos.201244)
Supplement: Supplementary Tables [file rsos201244supp1.docx]

**Supplementary Tables (Zäske, Skuk, and Schweinberger: Attractiveness and distinctiveness between speakers´ voices in naturalistic speech and their faces are uncorrelated)**

Table S1. Spearman ρ correlation coefficients (and p-values, uncorrected) for within-domain (voice and face) correlations between attractiveness and distinctiveness ratings (DITC in Exp.1 and 3; DEV in Exp. 2 and 4) depicted separately for female, male and all voices and faces in Exp. 1 - 4. Note that the strong and negative correlations between attractiveness and DEV-based distinctiveness (Exp. 2 and 4) survived Bonferroni correction for the 48 tests depicted in Tables S1 and S2 (corrected alpha level = .001), with the exception of the correlation for male faces in Exp. 2. The only other correlation which failed to reach significance after Bonferroni correction was the small positive correlation between voice attractiveness and DITC across all speakers (Exp. 3).

|  | Vowels | | | | |  | Sentences | | | | |
| --- | --- | --- | --- | --- | --- | --- | --- | --- | --- | --- | --- |
| Speaker Sex | Voices | |  | Faces | |  | Voices | |  | Faces | |
|  | Experiment 1 (DITC) | | | | |  | Experiment 3 (DITC) | | | | |
| Female | -.412 | (.019) |  | .229 | (.207) |  | .432 | (.013) |  | -.071 | (.698) |
| Male | -.324 | (.070) |  | .128 | (.484) |  | .119 | (.515) |  | -.089 | (.628) |
| **All** | **-.450** | **(< .001)** |  | **.207** | **(.101)** |  | **.275** | **(.028)** |  | **-.056** | **(.660)** |
|  | Experiment 2 (DEV) | | | | |  | Experiment 4 (DEV) | | | | |
| Female | -.842 | (< .001) |  | -.804 | (< .001) |  | -.906 | (< .001) |  | -.939 | (< .001) |
| Male | -.860 | (< .001) |  | -.496 | (.004) |  | -.804 | (< .001) |  | -.580 | (.001) |
| **All** | **-.853** | **(< .001)** |  | **-.639** | **(< .001)** |  | **-.866** | **(< .001)** |  | **-.744** | **(< .001)** |

Table S2. Spearman ρ correlation coefficients (and p-values, uncorrected) for between-domain (voice-face) correlations depicted separately for attractiveness and distinctiveness ratings (DITC in Exp.1 and 3; DEV in Exp. 2 and 4) of male, female and all speakers in Exp. 1 - 4. Note that the only significant correlation for attractiveness in vowels (Exp. 1) did not survive Bonferroni correction for the 48 tests depicted in Tables S1 and S2 (corrected alpha level = .001).

|  | Vowels | | | | |  | Sentences | | | | |
| --- | --- | --- | --- | --- | --- | --- | --- | --- | --- | --- | --- |
| Speaker Sex | Attractiveness | |  | Distinctiveness | |  | Attractiveness | |  | Distinctiveness | |
|  | Experiment 1 (DITC) | | | | |  | Experiment 3 (DITC) | | | | |
| Female | .191 | (.296) |  | -.016 | (.930) |  | -.078 | (.673) |  | .328 | (.067) |
| Male | .267 | (.140) |  | -.245 | (.177) |  | .294 | (.286) |  | -.076 | (.677) |
| **All** | **.304** | **(.014)** |  | **-.211** | **(.095)** |  | **.142** | **(.263)** |  | **.137** | **(.280)** |
|  | Experiment 2 (DEV) | | | | |  | Experiment 4 (DEV) | | | | |
| Female | .221 | (.225) |  | .224 | (.219) |  | -.052 | (.775) |  | .026 | (.886) |
| Male | .084 | (.648) |  | .049 | (.791) |  | .119 | (.516) |  | .153 | (.405) |
| **All** | **.237** | **(.059)** |  | **.184** | **(.145)** |  | **.091** | **(.476)** |  | **.091** | **(.476)** |

Table S3. Cronbach’s alpha for voice and face attractiveness and distinctiveness ratings in Exp. 1 - 4.

| Rating Dimension | Voices |  | Faces |  | Voices |  | Faces |
| --- | --- | --- | --- | --- | --- | --- | --- |
|  | Exp. 1 (vowels) | | |  | Exp. 3 (sentences) | | |
| Attractiveness | .845 |  | .899 |  | .886 |  | .899 |
| Distinctiveness (DITC) | .640 |  | .647 |  | .848 |  | .763 |
|  | Exp. 2 (vowels) | | |  | Exp. 4 (sentences) | | |
| Attractiveness | .892 |  | .893 |  | .820 |  | .885 |
| Distinctiveness (DEV) | .853 |  | .808 |  | .923 |  | .902 |

| Exp. 1 | Distinctiveness (VITC) | | | | | | | | | | | | | |
| --- | --- | --- | --- | --- | --- | --- | --- | --- | --- | --- | --- | --- | --- | --- |
| Attractiveness | /a/ | |  | /e/ | |  | /i/ | |  | /o/ | |  | /u/ | |
| /a/ | -.19 | (.128) |  | -.25 | (.042) |  | -.29 | (.020) |  | -.32 | (.010) |  | -.31 | (.012) |
| /e/ | -.02 | (.861) |  | -.40 | (.001) |  | -.11 | (.397) |  | -.22 | (.086) |  | -.24 | (.060) |
| /i/ | -.17 | (.190) |  | -.31 | (.012) |  | -.30 | (.014) |  | -.37 | (.003) |  | -.22 | (.084) |
| /o/ | .06 | (.661) |  | -.09 | (.479) |  | -.15 | (.246) |  | -.33 | (.008) |  | -.35 | (.005) |
| /u/ | .01 | (.967) |  | -.17 | (.187) |  | .02 | (.883) |  | -.28 | (.027) |  | -.17 | (.170) |

Table S4. Spearman ρ correlation coefficients (and p-values, uncorrected) for inter-vowel correlations between voice attractiveness and distinctiveness (VITC) ratings in Exp. 1.

| Exp. 2 | Distinctiveness (DEV) | | | | | | | | | | | | | |
| --- | --- | --- | --- | --- | --- | --- | --- | --- | --- | --- | --- | --- | --- | --- |
| Attractiveness | /a/ | |  | /e/ | |  | /i/ | |  | /o/ | |  | /u/ | |
| /a/ | -.74 | (< .001) |  | -.35 | (.005) |  | -.50 | (< .001) |  | -.40 | (.001) |  | -.33 | (.008) |
| /e/ | -.27 | (.034) |  | -.79 | (< .001) |  | -.50 | (< .001) |  | -.38 | (.002) |  | -.34 | (.007) |
| /i/ | -.51 | (< .001) |  | -.52 | (< .001) |  | -.79 | (< .001) |  | -.42 | (< .001) |  | -.35 | (.005) |
| /o/ | -.33 | (.007) |  | -.27 | (.033) |  | -.42 | (< .001) |  | -.60 | (< .001) |  | -.45 | (< .001) |
| /u/ | -.41 | (< .001) |  | -.25 | (.047) |  | -.41 | (< .001) |  | -.49 | (< .001) |  | -.57 | (< .001) |

Table S5. Spearman ρ correlation coefficients (and p-values, uncorrected) for inter-vowel correlations between voice attractiveness and distinctiveness (DEV) ratings in Exp. 2.

Table S6. Spearman ρ correlation coefficients (and p-values, uncorrected) for inter-sentence correlations between voice attractiveness and distinctiveness (VITC) ratings in Exp. 3.

| Exp. 3 | Distinctiveness (DITC) | | | | | | | |
| --- | --- | --- | --- | --- | --- | --- | --- | --- |
| Attractiveness | sentence #1 | |  | sentence #2 | |  | sentence #3 | |
| sentence #1 | .11 | (.389) |  | .07 | (.575) |  | -.07 | (.568) |
| sentence #2 | .28 | (.027) |  | .31 | (.014) |  | .11 | (.399) |
| sentence #3 | .41 | (< .001) |  | .39 | (.002) |  | .24 | (.059) |

Table S7. Spearman ρ correlation coefficients (and p-values, uncorrected) for inter-sentence correlations between voice attractiveness and distinctiveness (DEV) ratings in Exp. 4.

| Exp. 4 | Distinctiveness (DEV) | | | | | | | |
| --- | --- | --- | --- | --- | --- | --- | --- | --- |
| Attractiveness | sentence #1 | |  | sentence #2 | |  | sentence #3 | |
| sentence #1 | -.88 | (< .001) |  | -.60 | (< .001) |  | -.51 | (< .001) |
| sentence #2 | -.68 | (< .001) |  | -.85 | (< .001) |  | -.64 | (< .001) |
| sentence #3 | -.45 | (< .001) |  | -.57 | (< .001) |  | -.75 | (< .001) |

Table S8. Spearman ρ correlation coefficients (and p-values, uncorrected) for between-domain (vowels-face) correlations for attractiveness ratings in Exp. 1 and 2.

|  | Face Attractiveness | | | | |
| --- | --- | --- | --- | --- | --- |
| Voice  Attractiveness | Exp. 1 | |  | Exp. 2 | |
| /a/ | .28 | (.027) |  | .21 | (.101) |
| /e/ | .14 | (.284) |  | .21 | (.098) |
| /i/ | .23 | (.068) |  | .10 | (.418) |
| /o/ | .20 | (.122) |  | .06 | (.617) |
| /u/ | .14 | (.273) |  | .13 | (.316) |

Table S9. Spearman ρ correlation coefficients (and p-values, uncorrected) for between-domain (vowels-face) correlations for distinctiveness ratings in Exp. 1 (DITC) and 2 (DEV).

|  | Face Distinctiveness | | | | | |
| --- | --- | --- | --- | --- | --- | --- |
| Voice  Distinctiveness | Exp. 1  (DITC) | |  | Exp. 2  (DEV) | |  |
| /a/ | -.09 | (.491) |  | .01 | (.927) |  |
| /e/ | .02 | (.880) |  | .17 | (.191) |  |
| /i/ | -.06 | (.660) |  | .11 | (.367) |  |
| /o/ | -.26 | (.037) |  | .18 | (.154) |  |
| /u/ | -.21 | (.093) |  | .13 | (.295) |  |

Table S10. Spearman ρ correlation coefficients (and p-values, uncorrected) for between-domain (sentences-face) correlations for attractiveness ratings in Exp. 3 and 4.

|  | Face Attractiveness | | | | |
| --- | --- | --- | --- | --- | --- |
| Voice  Attractiveness | Exp. 3 | |  | Exp. 4 | |
| sentence #1 | .20 | (.117) |  | .20 | (.108) |
| sentence #2 | .10 | (.422) |  | .07 | (.579) |
| sentence #3 | .18 | (.164) |  | .05 | (.717) |

Table S11. Spearman ρ correlation coefficients (and p-values, uncorrected) for between-domain (sentences-face) correlations for distinctiveness ratings in Exp. 3 (DITC) and 4 (DEV).

|  | Face Distinctiveness | | | | |
| --- | --- | --- | --- | --- | --- |
| Voice  Distinctiveness | Exp. 3 (DITC) | |  | Exp. 4 (DEV) | |
| sentence #1 | .13 | (.294) |  | .11 | (.376) |
| sentence #2 | .05 | (.707) |  | .09 | (.475) |
| sentence #3 | .18 | (.157) |  | .05 | (.723) |

Table S12. Female listeners: Spearman ρ correlation coefficients (and p-values, uncorrected) for within-domain (voice and face) correlations between attractiveness and distinctiveness ratings (DITC in Exp.1 and 3; DEV in Exp. 2 and 4) depicted separately for female, male and all voices and faces in Exp. 1 – 4.

|  | Vowels | | | | |  | Sentences | | | | |
| --- | --- | --- | --- | --- | --- | --- | --- | --- | --- | --- | --- |
| Speaker Sex | Voices | |  | Faces | |  | Voices | |  | Faces | |
|  | Experiment 1 (DITC) | | | | |  | Experiment 3 (DITC) | | | | |
| Female | -.226 | (.213) |  | .257 | (.155) |  | .522 | (.002) |  | .060 | (.744) |
| Male | -.170 | (.351) |  | .041 | (.822) |  | .279 | (.122) |  | .151 | (.410) |
| **All** | **-.219** | **(.082)** |  | **.165** | **(.101)** |  | **.361** | **(.003)** |  | **.108** | **(.396)** |
|  | Experiment 2 (DEV) | | | | |  | Experiment 4 (DEV) | | | | |
| Female | -.887 | (< .001) |  | -.713 | (< .001) |  | -.791 | (< .001) |  | -.737 | (< .001) |
| Male | -.821 | (< .001) |  | -.350 | (.049) |  | -.858 | (< .001) |  | -.546 | (.001) |
| **All** | **-.866** | **(< .001)** |  | **-.525** | **(< .001)** |  | **-.821** | **(< .001)** |  | **-.518** | **(< .001)** |

Table S13. Male listeners: Spearman ρ correlation coefficients (and p-values, uncorrected) for within-domain (voice and face) correlations between attractiveness and distinctiveness ratings (DITC in Exp.1 and 3; DEV in Exp. 2 and 4) depicted separately for female, male and all voices and faces in Exp. 1 – 4.

|  | Vowels | | | | |  | Sentences | | | | |
| --- | --- | --- | --- | --- | --- | --- | --- | --- | --- | --- | --- |
| Speaker Sex | Voices | |  | Faces | |  | Voices | |  | Faces | |
|  | Experiment 1 (DITC) | | | | |  | Experiment 3 (DITC) | | | | |
| Female | -.406 | (.021) |  | .152 | (.405) |  | .224 | (.218) |  | -.073 | (.693) |
| Male | -.093 | (.614) |  | .229 | (.207) |  | .131 | (.476) |  | -.195 | (.284) |
| **All** | **-.406** | **(.001)** |  | **.205** | **(.104)** |  | **.193** | **(.126)** |  | **-.136** | **(.284)** |
|  | Experiment 2 (DEV) | | | | |  | Experiment 4 (DEV) | | | | |
| Female | -.534 | (.002) |  | -.712 | (< .001) |  | -.879 | (< .001) |  | -.892 | (< .001) |
| Male | -.821 | (< .001) |  | -.629 | (< .001) |  | -.783 | (< .001) |  | -.533 | (.002) |
| **All** | **-.681** | **(< .001)** |  | **-.627** | **(< .001)** |  | **-.836** | **(< .001)** |  | **-.743** | **(< .001)** |

Table S14. Female listeners: Spearman ρ correlation coefficients (and p-values, uncorrected) for between-domain (voice-face) correlations depicted separately for attractiveness and distinctiveness ratings (DITC in Exp.1 and 3; DEV in Exp. 2 and 4) of male, female and all speakers in Exp. 1 - 4.

|  | Vowels | | | | |  | Sentences | | | | |
| --- | --- | --- | --- | --- | --- | --- | --- | --- | --- | --- | --- |
| Speaker Sex | Attractiveness | |  | Distinctiveness | |  | Attractiveness | |  | Distinctiveness | |
|  | Experiment 1 (DITC) | | | | |  | Experiment 3 (DITC) | | | | |
| Female | .168 | (.357) |  | -.130 | (.479) |  | .019 | (.919) |  | .205 | (.260) |
| Male | .141 | (.441) |  | -.298 | (.097) |  | .191 | (.294) |  | .024 | (.895) |
| **All** | **.146** | **(.250)** |  | **-.242** | **(.054)** |  | **.294** | **(.018)** |  | **.089** | **(.482)** |
|  | Experiment 2 (DEV) | | | | |  | Experiment 4 (DEV) | | | | |
| Female | .164 | (.370) |  | .260 | (.150) |  | .007 | (.971) |  | .182 | (.318) |
| Male | -.104 | (.571) |  | .065 | (.725) |  | .329 | (.066) |  | .022 | (.904) |
| **All** | **.155** | **(.222)** |  | **.240** | **(.056)** |  | **.217** | **(.084)** |  | **.146** | **(.249)** |

Table S15. Male listeners: Spearman ρ correlation coefficients (and p-values, uncorrected) for between-domain (voice-face) correlations depicted separately for attractiveness and distinctiveness ratings (DITC in Exp.1 and 3; DEV in Exp. 2 and 4) of male, female and all speakers in Exp. 1 - 4.

|  | Vowels | | | | |  | Sentences | | | | |
| --- | --- | --- | --- | --- | --- | --- | --- | --- | --- | --- | --- |
| Speaker Sex | Attractiveness | |  | Distinctiveness | |  | Attractiveness | |  | Distinctiveness | |
|  | Experiment 1 (DITC) | | | | |  | Experiment 3 (DITC) | | | | |
| Female | .186 | (.309) |  | .078 | (.673) |  | -.149 | (.415) |  | .410 | (.020) |
| Male | .456 | (.009) |  | -.239 | (.187) |  | .178 | (.330) |  | -.262 | (.148) |
| **All** | **.309** | **(.013)** |  | **-.142** | **(.264)** |  | **.016** | **(.899)** |  | **.063** | **(.623)** |
|  | Experiment 2 (DEV) | | | | |  | Experiment 4 (DEV) | | | | |
| Female | .145 | (.428) |  | .099 | (.590) |  | -.035 | (.848) |  | -.120 | (.512) |
| Male | .264 | (.144) |  | .143 | (.433) |  | .195 | (.284) |  | .353 | (.048) |
| **All** | **.204** | **(.105)** |  | **.149** | **(.239)** |  | **.072** | **(.572)** |  | **.077** | **(.545)** |

Table S16. Spearman ρ correlation coefficients (and p-values, uncorrected) for within-domain (voice and face) correlations between attractiveness and distinctiveness ratings (DITC in Exp.1 and 3; DEV in Exp. 2 and 4) depicted separately for female, male and all voices and faces in Exp. 1 – 4, as well as comparisons between correlations for female vs. male stimuli according to [1], with Fisher’s Z (and p-values) in bold print.

|  | Vowels | | | | |  | Sentences | | | | |
| --- | --- | --- | --- | --- | --- | --- | --- | --- | --- | --- | --- |
| Speaker Sex | Voices | |  | Faces | |  | Voices | |  | Faces | |
|  | Experiment 1 (DITC) | | | | |  | Experiment 3 (DITC) | | | | |
| Female | -.412 | (.019) |  | .229 | (.207) |  | .432 | (.013) |  | -.071 | (.698) |
| Male | -.324 | (.070) |  | .128 | (.484) |  | .119 | (.515) |  | -.089 | (.628) |
| All | -.450 | (< .001) |  | .207 | (.101) |  | .275 | (.028) |  | -.056 | (.660) |
| **Female vs. Male** | **-0.388** | **( .698)** |  | **0.398** | **(.691)** |  | **1.305** | **(.192)** |  | **-0.069** | **(.945)** |
|  | Experiment 2 (DEV) | | | | |  | Experiment 4 (DEV) | | | | |
| Female | -.842 | (< .001) |  | -.804 | (< .001) |  | -.906 | (< .001) |  | -.939 | (< .001) |
| Male | -.860 | (< .001) |  | -.496 | (.004) |  | -.804 | (< .001) |  | -.580 | (.001) |
| All | -.853 | (< .001) |  | -.639 | (< .001) |  | -.866 | (< .001) |  | -.744 | (< .001) |
| **Female vs. Male** | **0.248** | **( .804)** |  | **-2.155** | **(.031)** |  | **-1.504** | **(.133)** |  | **-4.063** | **(< .001)** |

Table S17. Spearman ρ correlation coefficients (and p-values, uncorrected) for between-domain (voice-face) correlations depicted separately for attractiveness and distinctiveness ratings (DITC in Exp.1 and 3; DEV in Exp. 2 and 4) of male, female and all speakers in Exp. 1 - 4, as well as comparisons between correlations for female vs. male stimuli according to [1], with Fisher’s Z (and p-values) in bold print.

|  | Vowels | | | | |  | Sentences | | | | |
| --- | --- | --- | --- | --- | --- | --- | --- | --- | --- | --- | --- |
| Speaker Sex | Attractiveness | |  | Distinctiveness | |  | Attractiveness | |  | Distinctiveness | |
|  | Experiment 1 (DITC) | | | | |  | Experiment 3 (DITC) | | | | |
| Female | .191 | (.296) |  | -.016 | (.930) |  | -.078 | (.673) |  | .328 | (.067) |
| Male | .267 | (.140) |  | -.245 | (.177) |  | .294 | (.286) |  | -.076 | (.677) |
| All | .304 | (.014) |  | -.211 | (.095) |  | .142 | (.263) |  | .137 | (.280) |
| **Female vs. Male** | **-0.306** | **( .760)** |  | **0.891** | **(.372)** |  | **-1.451** | **(.147)** |  | **1.587** | **(.113)** |
|  | Experiment 2 (DEV) | | | | |  | Experiment 4 (DEV) | | | | |
| Female | .221 | (.225) |  | .224 | (.219) |  | -.052 | (.775) |  | .026 | (.886) |
| Male | .084 | (.648) |  | .049 | (.791) |  | .119 | (.516) |  | .153 | (.405) |
| All | .237 | (.059) |  | .184 | (.145) |  | .091 | (.476) |  | .091 | (.476) |
| **Female vs. Male** | **0.535** | **( .593)** |  | **0.681** | **(.496)** |  | **-0.654** | **(.513)** |  | **-0.488** | **(.625)** |

Table S18. Means and SD of vowel F0 (in Hz) in Exp. 5, collapsed across /a/ and /i/.

| Spreaker Sex | RecMode | Mean | SD |
| --- | --- | --- | --- |
| female | no model | 211 | 15 |
|  | model | 212 | 5 |
| male | no model | 112 | 16 |
|  | model | 114 | 15 |
| all | no model | 168 | 52 |
|  | model | 170 | 50 |

Table S19. Means and SD of sentence duration (in ms) in Exp. 5, collapsed across S1 and S2.

| Speaker Sex | RecMode | Mean | SD |
| --- | --- | --- | --- |
| female | no model | 1918 | 181 |
|  | model | 2124 | 165 |
| male | no model | 1906 | 172 |
|  | model | 2116 | 200 |
| all | no model | 1913 | 173 |
|  | model | 2121 | 176 |

Table S20. Means and SD of sentence F0 (in Hz) in Exp. 5, collapsed across S1 and S2.

| Speaker Sex | RecMode | Mean | SD |
| --- | --- | --- | --- |
| female | no model | 202 | 18 |
|  | model | 207 | 13 |
| male | no model | 115 | 18 |
|  | model | 113 | 15 |
| all | no model | 164 | 47 |
|  | model | 167 | 49 |

References

1 Diedenhofen, B., Musch, J. 2015 cocor: A Comprehensive Solution for the Statistical Comparison of Correlations. *Plos One*. **10**, (10.1371/journal.pone.0121945)
